# Amitriptyline for pain control in scleritis may help to avoid excessive steroid use in selected cases: a case report

**DOI:** 10.4076/1757-1626-2-8148

**Published:** 2009-08-13

**Authors:** Mya Thida Ohn, Deepak Gupta, Ben J Burton

**Affiliations:** Department of Ophthalmology, James Paget University Hospital NHS Foundation TrustLowestoft Road, Gorleston Norfolk, NR31 6LAUK

## Abstract

**Introduction:**

Pain in scleritis may be difficult to control with conventional immuno-suppressive regimes. Amitriptyline may be an effective option in those circumstances.

**Case presentation:**

A 41-year-old woman presented with scleritis and eye pain that was refractory to treatment with steroid and immunosuppressive agents. Effective pain relief and reduction of steroid dose was only achieved after starting amitriptyline.

**Conclusion:**

Amitriptyline may be an effective option for pain control in case with scleritis which might otherwise require unnecessarily large doses of immunosuppression.

## Introduction

Scleritis may cause pain that is refractory to conventional treatment with corticosteroids and immunosuppressive agents. Amitriptyline, a tricyclic anti-depressant sometimes used for neuropathic pain [1], may be an effective option for pain control in those cases of scleritis.

## Case presentation

A 41-year-old Caucasian woman was referred to eye casualty with a three-day history of a painful, red left eye. She had had similar episodes of bilateral anterior scleritis four years ago controlled by intravenous steroids and oral cyclosporin but had been off all immuno-suppression therapy for over three years. She was treated with oral flurbiprofen 100 mg t.d.s. This had no effect and after 48 hours she was commenced on 80 mg oral prednisolone. Two days later visual acuity reduced to 6/36 and she was placed on intravenous methylprednisolone 500 mg o.d. for three days. Haematological investigations excluded any systemic involvement. Ultrasound scan confirmed scleritis and MRI scan revealed no inflammatory orbital masses.

The inflammation and pain gradually settled and visual acuity improved to 6/6. She was discharged on oral prednisolone 80 mg daily as a reducing course. At 60 mg daily, she relapsed and required intravenous methylprednisolone 1 g stat followed by 500 mg o.d. for one week to control her inflammation. She declined cyclophosphamide because of the risk of side effects. Mycophenolate was started at 500 mg o.d., slowly increasing to 1 g b.i.d.

She developed weight gain and steroid-induced diabetes. She was an irregular attender at clinic and despite efforts to wean her off steroids she kept increasing the dose in order to control her ocular pain using between 60 and 70 mg prednisolone per day. There was a clear mismatch between the amount of ocular inflammation clinically and her symptoms of pain. Five months after presentation a trial of oral amitriptyline was commenced at 10 mg nocte increasing to 50 mg nocte over 1 week. This very rapidly controlled the pain and she was able to reduce her steroid dose from 60 mg to 15 mg prednisolone daily within fifteen weeks of starting amitriptyline. At this point her pain returned but this time with clear clinical evidence of her scleritis becoming more active. This flare up was controlled by briefly increasing her prednisolone to 40 mg daily and her mycophenolate to 1.5 mg b.i.d. She is now on 10 mg prednisolone o.d., mycophenolate 1.5 mg b.i.d. and amitriptyline 50 mg nocte without further relapse.

## Discussion

Pain in scleritis may be very severe and can often wake the patient from sleep. Treatment of the inflammation is usually adequate to control the pain. In this case there was a clear mismatch between the visible signs of inflammation and the pain reported by the patient. We believe that it is important to recognise and distinguish between treating inflammation and treating pain in such cases to avoid excessive and unnecessary use of immunosuppressive drugs. A trial of amitriptyline, a tricyclic antidepressant, may be beneficial although it would also be perfectly reasonable to use other drugs such as gabapentin [2-4].

## Conclusion

We can find no report in the literature of amitriptyline used in this way for patients with scleritis and would recommend considering its use for the control of pain from scleritis where the pain is out of proportion to the inflammation.

## Abbreviation

MRI: magnetic resonance imaging
